# Complete ophthalmoplegia, complete ptosis and dilated pupil due to internal carotid artery dissection: as the first manifestation of Takayasu arteritis

**DOI:** 10.1186/s12872-017-0638-7

**Published:** 2017-07-25

**Authors:** H. M. M. T. B. Herath, S. P. Pahalagamage, D. Withana, Sunethra Senanayake

**Affiliations:** National Hospital, Colombo, Sri Lanka

**Keywords:** Complete ophthalmoplegia, Internal carotid artery dissection, Takayasu arteritis

## Abstract

**Background:**

Takayasu arteritis is a rare, chronic large vessel vasculitis involving the aorta and its primary branches. As the disease progresses, the active inflammation of large vessels leads to dilation, narrowing and occlusion of the arteries. Arterial dissection is due to separation of the layers of the arterial wall resulting in a false lumen, where blood seeps into the vessel wall. Neurological sequelae of intracranial arterial dissection results from cerebral ischemia due to thromboembolism and hypo perfusion. Internal carotid artery dissection in Takayasu arteritis is very rare and complete ophthalmoplegia due to internal carotid artery dissection is also rare. This is the first case report of Takayasu arteritis presenting as complete ophthalmoplegia due to internal carotid artery dissection.

**Case presentation:**

A 38-year-old Sri Lankan female presented with sudden onset severe headache, fixed dilated pupil, complete ptosis and ophthalmoplegia on the right side. On imaging, dissection and dilatation was evident in the right internal carotid artery from the origin up to the cavernous segment. She also had stenosis and aneurysmal dilatation of right subclavian artery. Takayasu arteritis was diagnosed subsequently. She was started on aspirin and high dose steroids.

**Conclusions:**

Internal carotid artery dissection within the cavernous sinus can lead to third, fourth and sixth nerve palsy due to compression, stretching and ischemia from occlusion of the nutritional arteries. This case report illustrates that internal carotid artery dissection should be a differential diagnosis in palsies of the third, fourth, or sixth cranial nerves, especially when associated with headache. In cases of internal carotid artery dissection, vasculitis such as Takayasu arteritis should also be considered.

## Background

Takayasu arteritis is a rare, chronic large vessel vasculitis involving the aorta and its primary branches. Females are affected more and the age of onset is usually between 10 and 40 years [1, 2]. As the disease progresses the active inflammation of large vessels leads to dilation, narrowing and occlusion of the arteries. Subclavian artery involvement is common and involvement of the carotid and vertebral arteries causes neurological symptoms such as headache, visual disturbance, strokes, transient ischemic attacks and seizures.

Arterial dissection is due to separation of the layers of an arterial wall resulting in a false lumen, where blood seeps into the vessel wall. Neurological sequelae of intracranial arterial dissection result from cerebral ischemia due to thromboembolism and hypo perfusion. In addition, dissection and aneurysmal dilatation of carotid arteries can cause neurological manifestations due compression of adjacent nerves and their feeding vessels resulting in pain, Horner syndrome and cranial neuropathies. Common causes for internal carotid artery (ICA) dissection include trauma, Ehlers–Danlos syndrome, Fibromuscular dysplasia, Marfan syndrome, cystic medial necrosis, autosomal dominant polycystic kidney disease and homocystinuria.

Here we describe a middle aged female who presented with sudden onset complete ptosis, complete ophthalmoplegia and fixed dilated pupil on the right side. On imaging dissection and dilatation was evident in the right ICA from the origin up to the cavernous segment. She also had stenosis and aneurysmal dilatation of right subclavian artery and Takayasu arteritis was diagnosed subsequently.

ICA dissection in Takayasu arteritis is very rare and complete ophthalmoplegia due to ICA dissection is also rare. This is the first case report of Takayasu arteritis presenting as complete ophthalmoplegia due to ICA dissection.

## Case presentation

A 38-year-old Sri Lankan female presented with sudden onset severe right side headache and vomiting. At the same time she developed right-sided complete ptosis and ophthlamoplegia. The headache and vomiting lasted for one day, but the ptosis and ophthalmoplegia persisted. She did not have a history of diabetes, hypertension, dyslipidemia or any connective tissue disorder.

On examination she had complete ptosis, ophthalmoplegia and a fixed dilated pupil (5 mm) on the right side. Direct and consensual light reflexes were absent on the same side. Vision and fundoscopic examination was normal. Left eye was normal with full eye movements and light reflexes. Other cranial nerves including sensory component of the trigeminal nerve were normal. She did not have scalp tenderness or tenderness over the superficial temporal artery. Upper limbs and lower limbs were neurologically normal and she did not have any cerebellar signs. Brachial pulse was decreased on the right side compared to left side. Blood pressure on the right arm was 90/ 60 mmHg and on the left arm it was 120/ 80 mmHg. She had bruit over right subclavian and carotid arteries. Rest of the cardiovascular, respiratory and abdominal examination was normal. She did not have features of Ehlers–Danlos syndrome, Marfan syndrome or manifestations of connective tissue disorder.

**Fig. 1:**
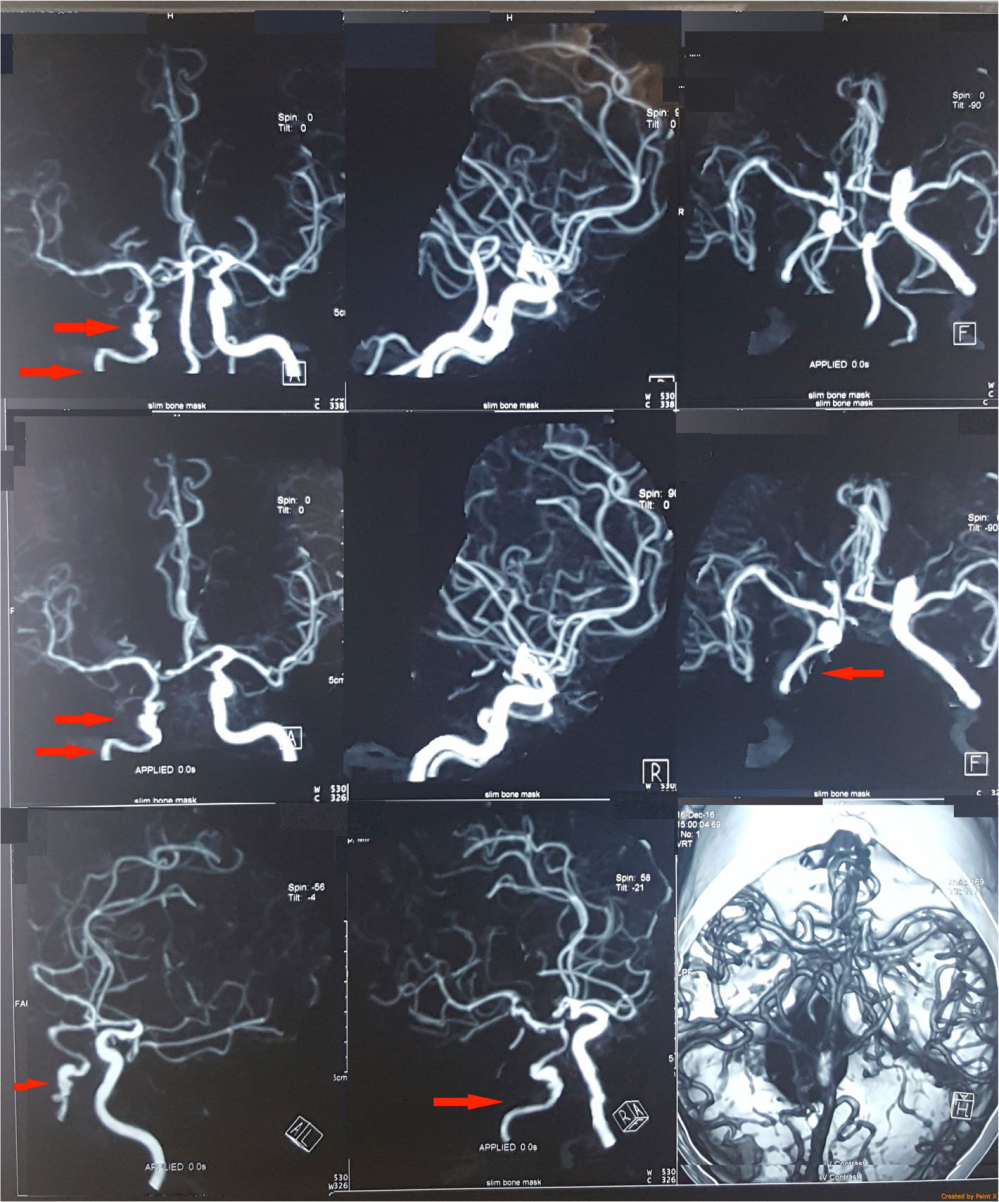
CT cerebral angiogram showing generalized caliber reduction of right internal carotid artery (Shown by *red arrow*)

**Fig. 2:**
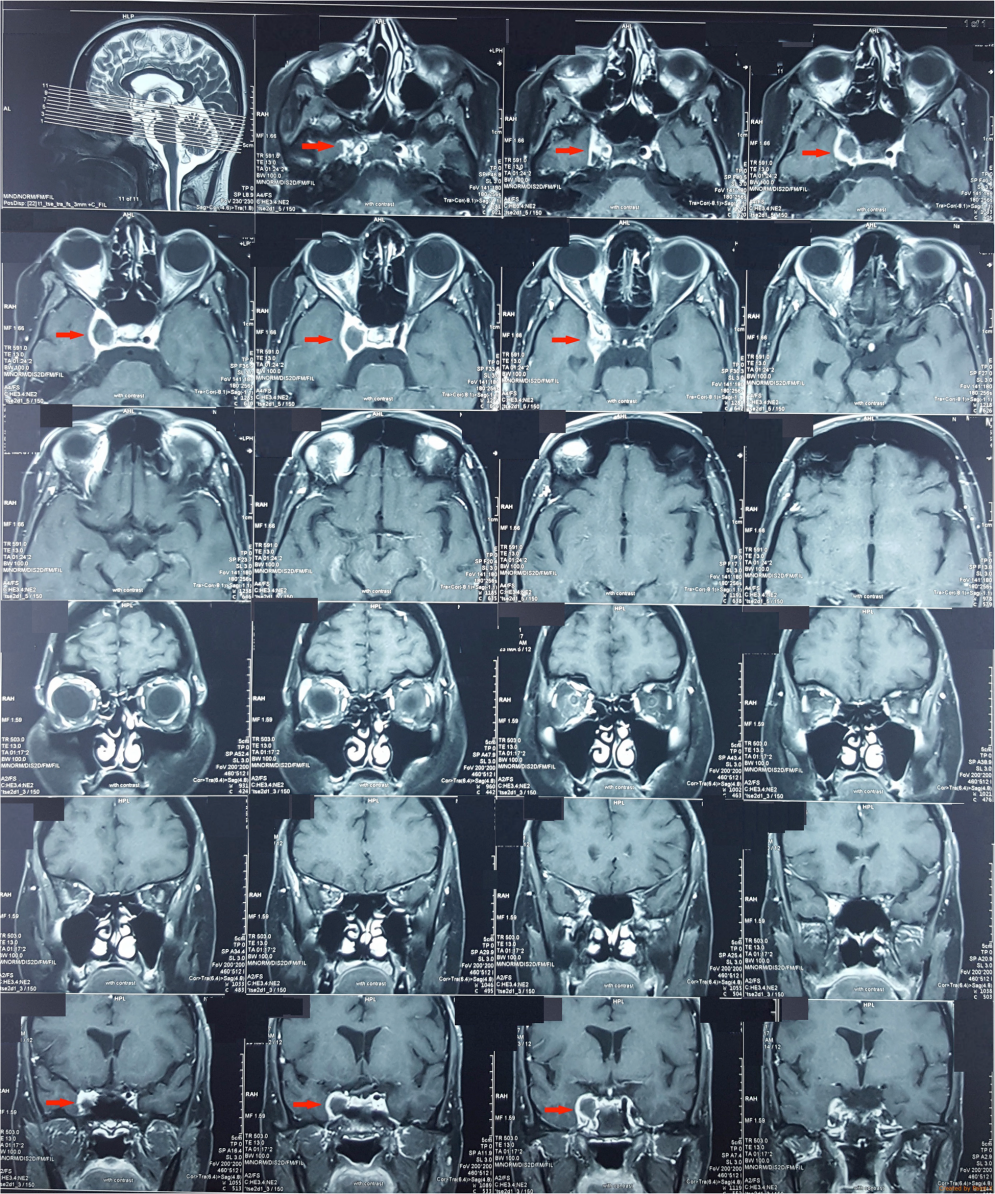
MRI showing dissection of the right internal carotid artery in the cavernous sinus (shown by the *red arrow*). A thrombosed false lumen of 1.5 × 1 cm, was seen. The thrombosed lumen was of intermediate signal intensity in and did not enhance with contrast. This was compressing the right cavernous sinus. Right internal carotid artery was significantly narrowed

**Fig. 3:**
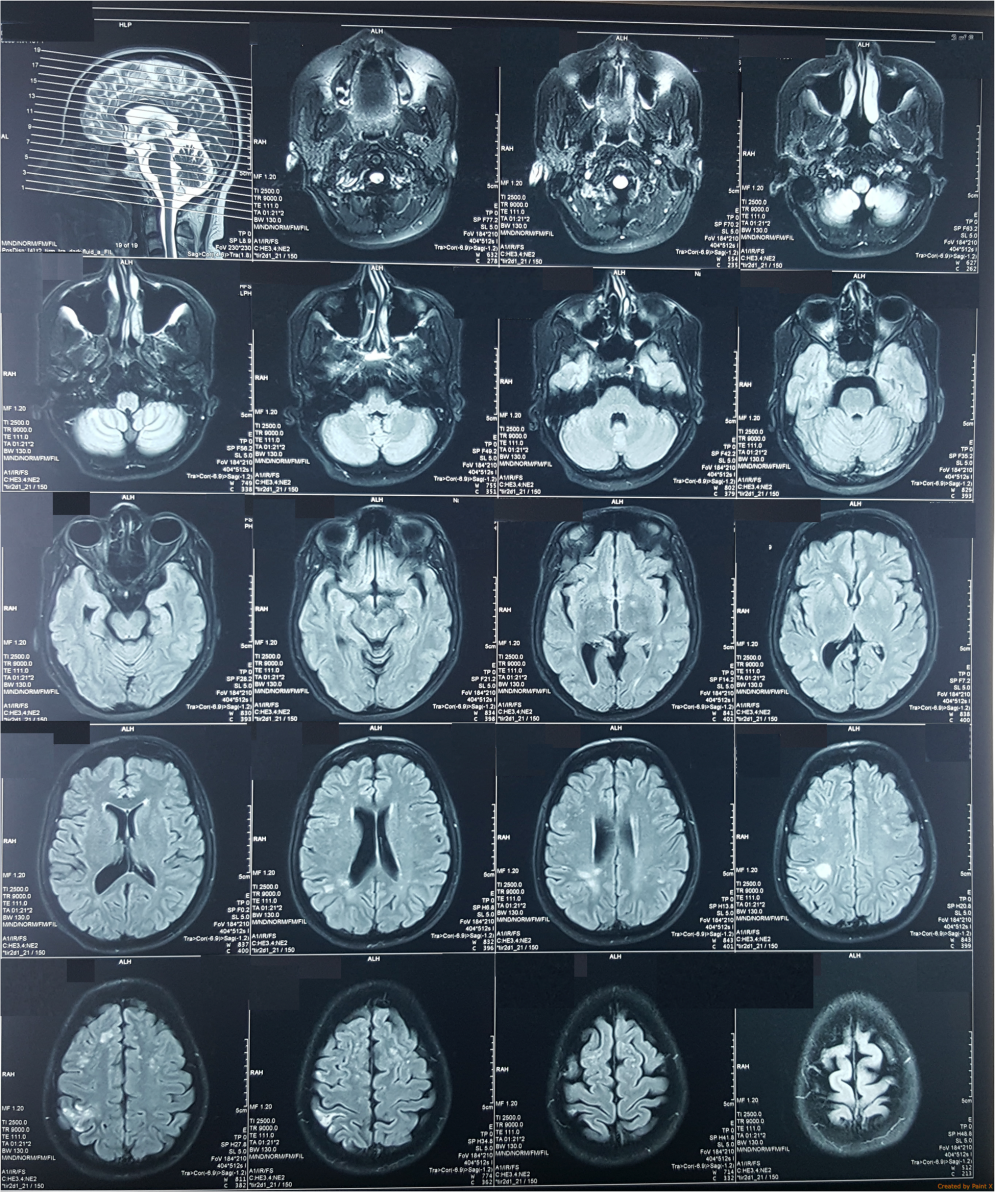
FLAIR images showing multiple, focal hyper intensities with partially restricted diffusion, suggestive of acute infarcts in the right parietal lobe

**Fig. 4:**
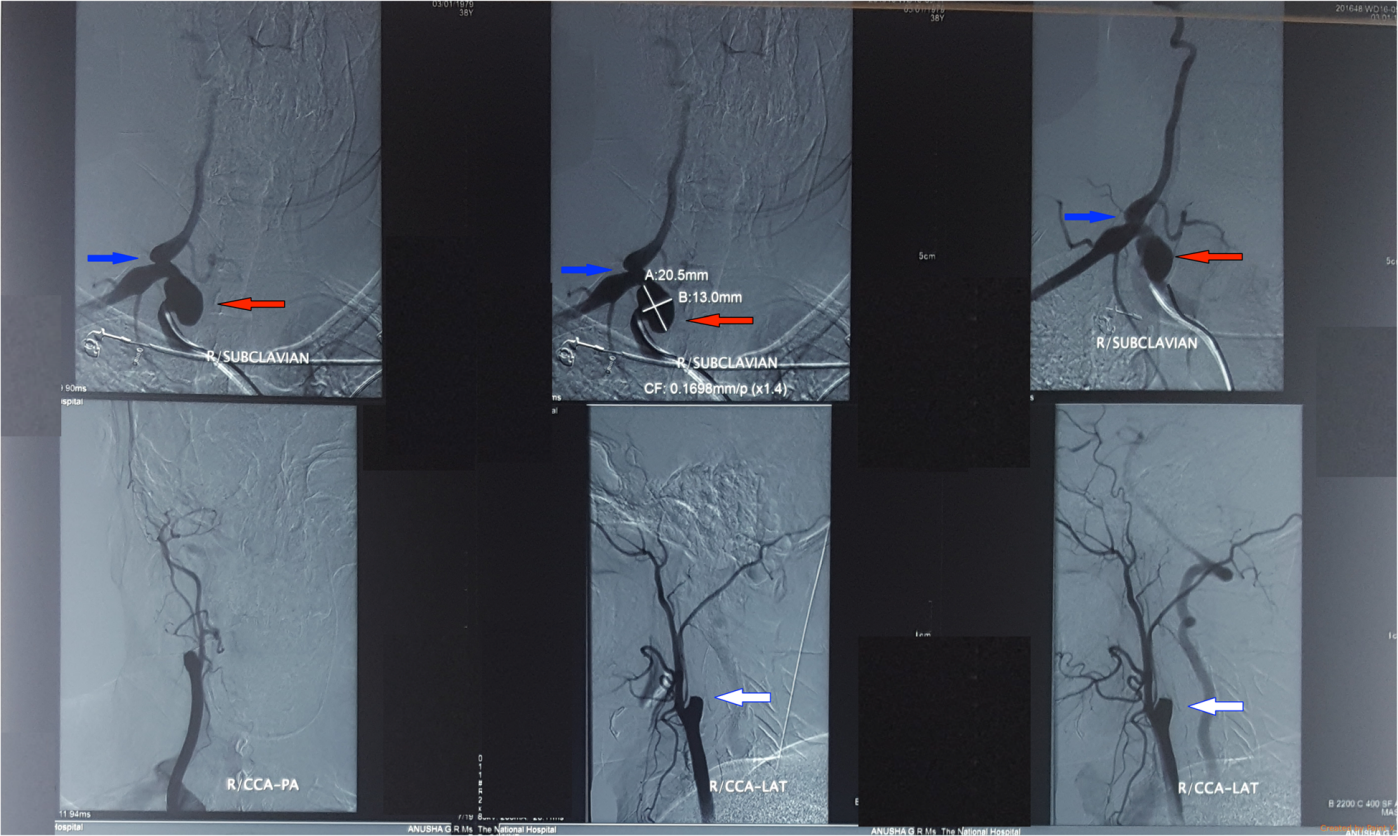
Cerebral digital subtraction angiogram showing total occlusion of the right internal carotid artery from its origin (indicated by *white arrow*). Aneurysmal dilation (*Red arrow*) and stenosis (*Blue arrow*) was also evident at the right proximal subclavian artery

**Fig. 5:**
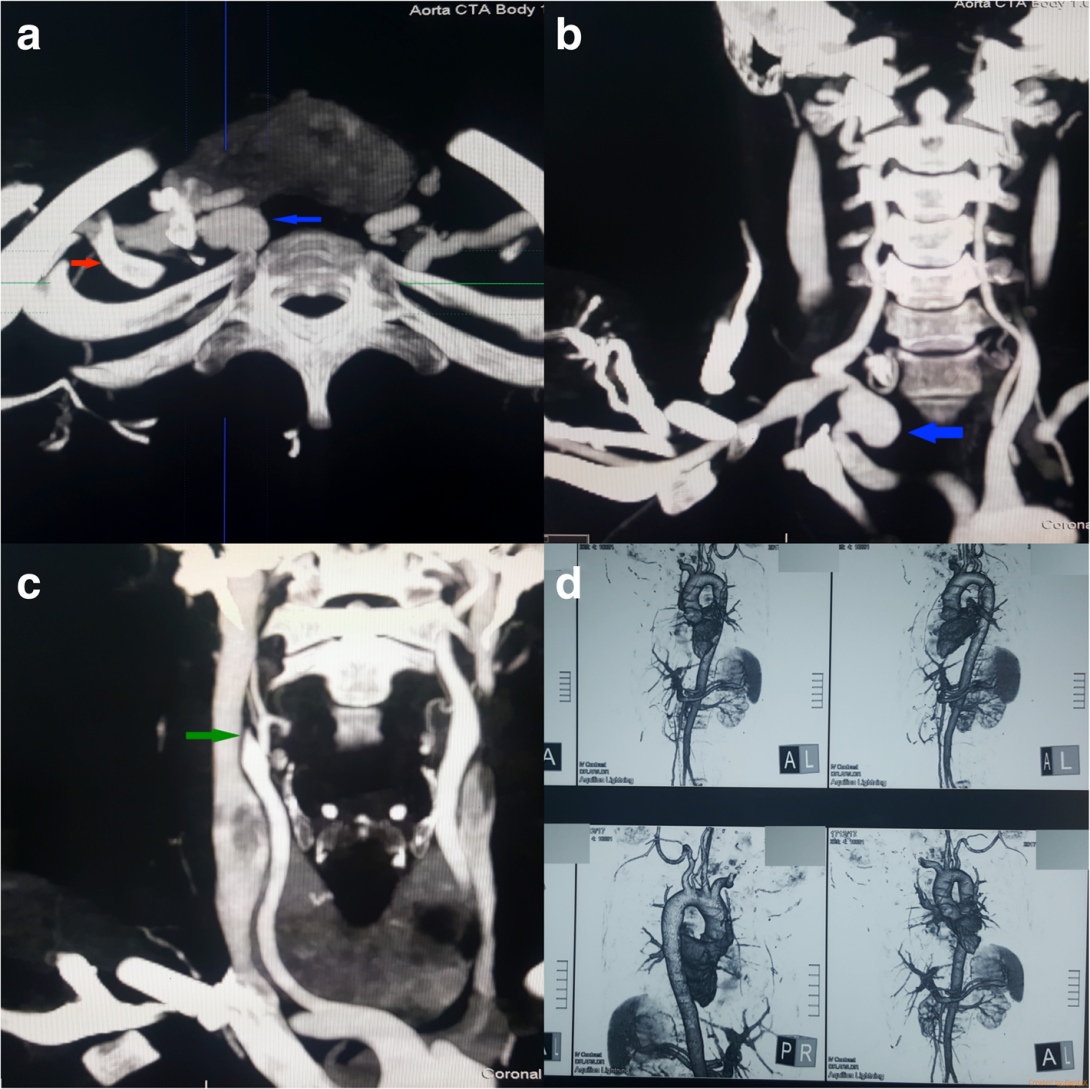
CT angiogram showing aneurismal dilatation (20 × 11 mm) of the first part of the right subclavian artery(in **a** and **b** shown by *blue arrow*). Tapering of the distal part of the right carotid bulb at the origin of the right internal carotid artery (shown by the *green arrow*) was suggestive of right internal carotid artery dissection with thrombosis. *Red arrow* showing the cervical rib. Rest of the angiogram was normal (Shown by **d**)

Noncontract CT brain was done soon after the admission and was normal. Cerebrospinal fluid analysis, performed on day 2 to exclude a subarachnoid heamorrhage, was normal with absent cells, normal protein level and did not reveal xanthochromia. CT cerebral angiogram (Fig. 1) done on day 4 revealed generalized caliber reduction of right ICA. MRI done on day 23 showed significantly narrowed right ICA. Dissection of the right ICA was noted in the cavernous sinus, with a false lumen of 1.5 × 1 cm, which was thrombosed (Fig. 2). The thrombosed lumen was of intermediate signal intensity in T1, T2 and Fluid-attenuated inversion recovery (FLAIR) images and did not enhance with contrast. This was compressing the right cavernous sinus. Multiple focal T2 and FLAIR hyper intensities with partially restricted diffusion, suggestive of acute infarcts, were also seen in the right parietal lobe (Fig. 3). Following this we performed a cerebral digital subtraction angiogram, which showed total occlusion of the right ICA from its origin (Fig. 4). Blood supply to the right middle cerebral artery was maintained via the anterior communicating and the posterior communicating arteries. Aneurysmal dilation and stenosis were also evident at the right proximal subclavian artery (Fig. 4). The CT angiogram also revealed aneurismal dilatation (20 × 11 mm) and stenosis of the first part of the right subclavian artery (Fig. 5). Tapering of the distal part of the right carotid bulb at the origin of the right ICA was seen and was suggestive of right ICA dissection with thrombosis. Rest of the angiogram was normal (Fig. 5). Florescent angiogram of the retina was normal.

Full blood count showed neutrophil leukocytosis with low hemoglobin and normochromic normocytic anemia was seen in the blood picture (Table 1). Liver function tests revealed low albumin with normal liver enzyme levels (Table 1). Serum creatinine was 150 μmol/L (eGFR using MDRD = 35.8 ml/min/ 1.73m^2^) on admission and later came down to 100 μmol/L (eGFR using MDRD = 57.2 ml/min/73m^2^). Urine full report was normal and ultrasound revealed the kidney sizes to be 8.9 cm on the right and 8.7 cm on the left. Ionized calcium was 1.11 mmol/L (1.0–1.3) and phosphorus was 1.0 mmol / L (0.8–1.5). ESR was persistently high (130 mm in the first hour) and CRP was 95 mg/L, which later came down to 7 mg/L. She was negative for HIV serology and VDRL was non reactive. Mantoux test was negative and the chest x ray was normal. ANA, p-ANCA, c-ANCA and hepatitis B and C serology were negative. Fasting blood sugar, HbA1C, thyroid function tests and lipid profile were within normal range. Bilateral cervical ribs were noted in chest x-ray and in cervical x- ray.

**Table 1:** Full blood count and liver function tests

Investigation and value	Normal range	Investigation and value	Normal range
WBC 13.65 × 103 /μL	4–10	Neutrophils 11.44× 103 /μL	2–7
Lymphocytes 1.36 × 103 /μL	0.8–4	Platelets 244 × 10^3^ /μL	150–450
Hemoglobin = 10.7 g/dL	11–16	RDW – CV 0.125	0.110–0.160
MCV 85.4 fL	80–100	MCH 27.5 pg	27–34
MCHC 31 g/dL	32–36		
AST 20 U/L	10–35	ALT 26 U/L	10–40
Alkaline phosphatase = 84 U/L	100–360	INR 1.26	
Albumin = 31 g/L	36–50	Globulin 28.0 g/L	22–40

*WBC* White blood cells, *RDW* Red cell distribution width, *MCV* Mean corpuscular volume, *MCH* Mean corpuscular hemoglobin, *MCHC* Mean corpuscular hemoglobin concentration, *AST* aspartate aminotransferase, *ALT* Alanine transaminase, *INR* international normalized ratio

Takayasu arteritis was diagnosed and she was started on high dose of prednisolone. (1 mg/kg). Aspirin was also started. Because of critical cerebral artery complication neurosurgical and vascular surgical opinion was taken. As the patient did not have any further neurological events and because of limited facilities available, no surgical intervention was planned. After 2 weeks of steroids her ESR came down to 50 mm in the first hour and after one month ESR was 30 mm in the first hour. Patient’s ophthalmoplegia and ptosis remained the same and we started to tail off steroids gradually and added azathioprine as a steroid-sparing agent. The difference between right and left brachial pulses reduced and the right arm blood pressure increased. She did not have any further vascular events and a follow up angiogram was planned in six months time. Authors followed the CARE guidelines when writing this case report.

## Discussion and conclusions

Diagnosis of Takayasu arteritis in this patient was made using The American College of Rheumatology criteria [2]. Her age was less than 40 years. She had low pulse volume and blood pressure in the right arm, bruit over the right subclavian and carotid arteries along with angiographic evidence of narrowing and aneurysm of right subclavian artery and dissection of right ICA. She did not have any risk factors for atherosclerosis, any evidence of fibro muscular dysplasia or other syndromic features that can explain large artery involvement. Her ESR was persistently high which was in favor of an inflammatory disease and antibody screening for other connective tissue disorders were negative. Even though cervical ribs were present, CT angiogram showed no association between those and the subclavian artery pathology. We also performed nerve conduction studies, which excluded C8/T1 nerve root compression, which is seen in thoracic outlet syndrome.

We concluded that she had a dissection of the right ICA because the history was acute onset with severe headache and the MRI and CT angiogram had evidence of arterial dissection. Later, the right ICA thrombosed completely and the DSA showed complete occlusion from the origin of the right ICA. Following the complete occlusion of the right ICA, blood supply to the right side of the brain was maintained via communicating arteries. Only small multiple infarcts in right parietal lobe were evident in the MRI, which were clinically silent.

ICA originates from the common carotid artery and Bouthillier described seven anatomical segments of the ICA [3]. The cavernous segment passes through the cavernous and is closely related to the third fourth and sixth cranial nerves. Ophthalmic and maxillary branches of the trigeminal nerve are also closely associated. It also gives off the inferolateral trunk, which supplies these cranial nerves [4, 5]. Complete ophthalmoplegia in our patient was thought to be due to compression and stretching of the third, fourth and sixth nerves at the cavernous sinus from the dissected ICA. Occlusion of the inferolateral trunk can also cause ischemic damage to these cranial nerves [5]. Surprisingly in our patient the trigeminal nerve was not affected. Pupillary involvement can also be explained by the damage to the parasympathetic fibers of the oculomotor nerve. The existence of Horner’s syndrome was difficult to determine since the patient had complete ptosis and mydriasis due to complete third nerve palsy. Involvement of the ophthalmic artery, the first branch of the ICA distal to the cavernous sinus, can lead to retinal ischemia and blindness but this was not seen in our patient and the fluorescent angiogram of the retina was normal.

Carotid artery dissection and aneurysmal dilatation can cause symptoms from compression of adjacent nerves and their feeding vessels. Bahram Mokri et al., stated that 23 of 190 (12%) with spontaneous dissection of the extra cranial ICA had cranial nerve palsies [6]. Majority of them had lower cranial nerve palsies and only a few had fifth and seventh nerve involvement, oculomotor palsies and ischemic optic neuropathy [6]. Ophthalmoplegia following ICA dissection is rarely reported in literature. Vargas, M.E. et al., reported a 29 year old patient with an ipsilateral ophthalmoplegia as the presentation of a traumatic dissection of the ICA [7]. He had complete ophthalmoplegia on the left side with partial ptosis and a fixed and dilated pupil. Reduced sensation of the first and second parts of the trigeminal nerve was also noted. However the vision and fundoscopy were normal. This presentation was similar to our patient except that our patient did not have fifth nerve involvement. Wilson, W.B. et al., reported 3 patients with unilateral ophthalmoparesis following acute thrombosis of the ICA and all had sudden monocular blindness [8]. Each had mild-to-moderate signs of contralateral hemispheric dysfunction and the thrombus had extended from the origin of the internal carotid to its intracranial bifurcation into the anterior and middle cerebral arteries. Proximal one-half to two-thirds of the ophthalmic artery was also occluded in those cases. Galetta, S.L., et al., reoprted a patient with traumatic bilateral carotid artery dissections who presented with acute loss of vision, proptosis, ophthalmoparesis, conjunctival injection and chemosis [9]. Schievink, W.I., et al., described four patients among 155 patients with spontaneous dissections of the cervical ICA who had third, fourth, or sixth cranial nerve palsy [10]. Three had oculosympathetic palsy and none had any associated cerebral or retinal ischemic symptoms. Campos, C.R., et al., reported a 50-year-old non-diabetic man who experienced acute onset right sided headache, impaired adduction and upward gaze of right eye and slight ipsilateral pupillary dilatation without ptosis. His angiography showed right ICA dissection with forward occlusion to the base of the skull [11].

Stenosis is the commonest cause leading to vascular manifestations in arteritis, and aneurysms and dissection is rare [12, 13]. We could only find 2 patients with Takayasu arteritis in the literature who had ICA dissection. Caso V., et al., described a young patient with right ICA dissection and later this patient met the diagnostic criteria for systemic lupus erythematosus, Takayasu arteritis and the antiphospholipid syndrome [14]. Another case report described a 35-year-old woman presenting with a left temporal headache, dysphasia and right hemiparesis, whose MRI of the brain revealed left middle cerebral (posterior branch) artery acute ischemia. Cervical vessel ultrasonography and digital subtraction angiography showed a left ICA dissection. A 2-year follow-up revealed 90% concentric, segmental right common carotid artery stenosis and persistent left ICA occlusion. Takayasu arteritis was diagnosed and steroids were started. Prosthetic replacement of the right common carotid artery was also performed [15].

The American Heart Association/American Stroke Association (AHA/ASA) guidelines in 2014 recommend the use of antithrombotic therapy for at least three to six months in carotid artery dissection. However, the relative efficacy of antiplatelet versus anticoagulation therapy is unknown [16]. Similarly National Institute for Health and Clinical Excellence recommend treatment with either anticoagulants or antiplatelet agents [17]. Therefore we started aspirin in our patient after the diagnosis of ICA dissection. The mainstay of therapy for Takayasu arteritis is glucocorticoids and we started high doses of prednisolone.

In conclusion, ICA dissection is a rare manifestation of Takayasu arteritis. ICA dissection within the cavernous sinus can lead to third, fourth and sixth nerve palsy due to compression and stretching of the nerves and ischemia resulting from compression of the nutritional arteries. Here we describe a patient who presented with complete ophthalmoplegia and fixed dilated pupil following ICA dissection in whom Takayasu arteritis was diagnosed. This case report illustrates that ICA dissection should be a differential diagnosis of palsies of the third, fourth, or sixth cranial nerves, especially when associated with headache. In cases of ICA dissection, vacuities such as Takayasu arteritis should also be considered.

## Abbreviations

ANA: Antinuclear autoantibodies
ANCA: Anti-neutrophil cytoplasmic antibodies
eGFR: estimated glomerular filtration rate
FLAIR: Fluid-attenuated inversion recovery
HIV: Human immunodeficiency virus
ICA: Internal carotid artery
MDRD: Modification of Diet in Renal Disease
MRI: Magnetic resonance imaging
VDRL: Venereal disease research laboratory
